# Feature-based behavior coding for efficient exploratory analysis using pose estimation

**DOI:** 10.3758/s13428-025-02702-6

**Published:** 2025-05-09

**Authors:** Eigo Nishimura

**Affiliations:** Department of Human Life Design and Science, Kyushu University, 4-9-1 Shiobaru, Minamiku, Fukuoka, 815-8540 Japan

**Keywords:** Open-source software, Behavioral coding, Video analysis, Keypoint detection, Observational methods

## Abstract

This paper introduces feature-based behavior coding (FBBC), an efficient method for exploratory analysis in behavioral research using pose estimation techniques. FBBC addresses the challenges of traditional behavioral coding methods, particularly in the exploratory stages of research when coding schemes are not yet well defined. By leveraging keypoint detection and dimensionality reduction, FBBC transforms video data into interpretable feature time series, enabling researchers to analyze diverse postural patterns more efficiently. Also presented is Behavior Senpai, an open-source software implementation of FBBC that integrates automated feature extraction with human insight. A case study demonstrates FBBC’s ability to classify complex postures by combining multiple features and manual clustering. While the current iteration focuses on instantaneous posture classification, the framework shows potential for expansion to action classification. FBBC offers increased flexibility in developing coding schemes and reduces the time-consuming nature of repetitive observations. This approach represents a considerable advancement in behavioral research, bridging traditional methods with modern machine-learning techniques. As FBBC is adopted and refined, it will contribute to more comprehensive and insightful behavioral analyses across the psychological and behavioral sciences.

## Introduction

### Challenges in traditional behavioral coding

Behavioral coding is a cornerstone of psychological and behavioral research, enabling systematic analyses of complex human behaviors (Bakeman & Quera, 2011). This method has been instrumental in a range of fields, from developmental psychology to clinical interventions (Chorney et al., 2015). Traditional coding methods typically involve repeated observational cycles, where researchers iteratively refine their questions and hypotheses through exploratory analysis (Bakeman & Quera, 2011). This iterative process allows for the gradual development of coding schemes but also presents significant challenges, particularly during exploratory research when coding schemes are not yet well defined. The most prominent of these challenges are the time-consuming and labor-intensive nature of video review and the difficulty in consistently identifying and coding diverse postural patterns across large datasets. While software tools such as Simple Video Coder (Barto et al., 2017) and Video Activity Coder (Braswell, 2021) enhance coding efficiency, they still depend heavily on manual, repetitive observation of video footage. As a result, they can be time-consuming and contribute to observer fatigue. As Nota et al. (2023) demonstrated in their study of facial signals and social actions, the complexity of human behavior often requires nuanced and flexible coding approaches. Moreover, as Linanza et al. (2024) showed in their exploratory study, traditional methods may struggle to capture the full richness of behavioral data, especially in complex social contexts.

### Advancements in behavioral analysis

Concurrently, advancements in machine learning and computer vision offer new possibilities for behavioral analysis. Techniques such as pose estimation, as demonstrated in DeepLabCut (Mathis et al., 2018), show promise for automating aspects of behavioral analysis. Unlike pretrained pose estimation models, DeepLabCut requires manual annotation of training data, which can be labor-intensive. This limitation is particularly pronounced when analyzing human behavior, as opposed to relatively planar movements in rodents, since human movements—especially hand gestures—exhibit greater variability, necessitating a greater volume of annotated training data. Furthermore, DeepLabCut itself is primarily designed for keypoint detection; more advanced behavioral classification and analysis typically require additional tools and methodologies. For instance, extracted keypoints require further processing, and kinematic analysis may be needed to derive motion parameters such as velocity, acceleration, or joint angles. The concept of quantifying behavior via specific features has a rich history in behavioral research. A pioneering example is the Facial Action Coding System developed by Ekman and Friesen (1978), in which facial expressions are broken down into discrete measurable units. This approach to feature-based behavior analysis is foundational for more advanced computational methods.

### Introducing feature-based behavior coding and Behavior Senpai

This paper introduces feature-based behavior coding (FBBC), an approach that bridges traditional behavioral coding and advanced machine-learning techniques. FBBC leverages keypoint detection to transform video data into interpretable formats while preserving the crucial role of human insight in the coding process, thereby addressing the need for flexible and efficient coding methods highlighted by Chorney et al. (2015). FBBC addresses key limitations of existing methods in several ways. First, it reduces the need for repetitive video reviewing via feature time-series conversion. Second, it provides a more holistic view of postural patterns across entire datasets. Finally, it maintains flexibility for exploratory research via iterative refinement of feature definitions and clustering approaches. To implement FBBC, we have developed Behavior Senpai (where “Senpai” is a Japanese term referring to a senior or mentor who guides a junior), an open-source software tool that integrates keypoint detection, feature extraction, and interactive clustering. This name reflects the tool's design philosophy: Behavior Senpai is not a fully automated analysis system but functions as a collaborative guide that combines computational capabilities with human insight. In this collaborative approach, the tool provides efficient data processing and visualization while leaving final interpretation and judgment to the researcher's expertise. This tool provides researchers with a user-friendly interface with which to apply FBBC to their video data, thereby enabling rapid exploration and analysis of behavioral patterns. In doing so, it builds upon the foundational work of Bakeman and Quera (2011) on sequential analysis and observational methods.

In the following sections, we detail FBBC’s theoretical foundations, describe Behavior Senpai’s implementation, and provide a case study demonstrating its application to a complex behavioral task. We also discuss the potential implications of this approach for behavioral research, its current limitations, and future development directions. FBBC and Behavior Senpai represent an innovative approach to behavioral coding, one that addresses the challenges of exploratory research while leveraging the strengths of human insight and computational analysis. This approach has the potential to advance behavioral research considerably, building on the rich history of observational methods while embracing the possibilities offered by modern technology.

## System technical overview

### Workflow diagram

**Fig. 1:**
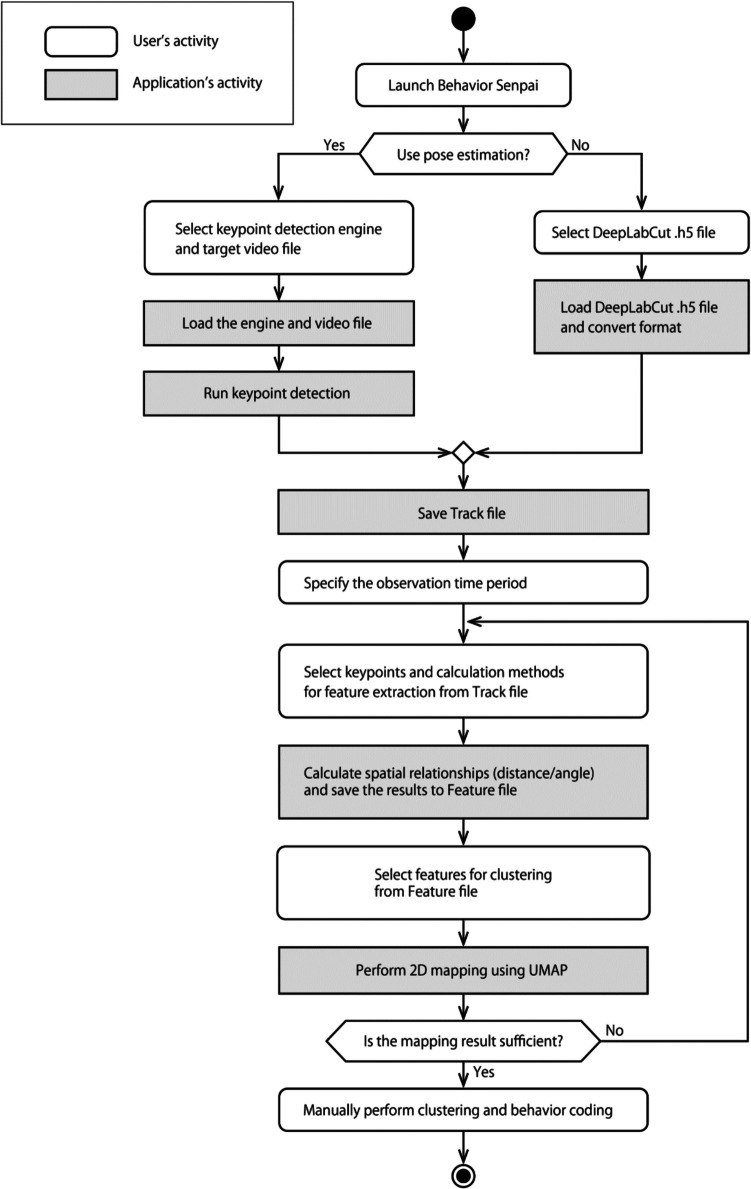
Activity diagram of Behavior Senpai

Behavior Senpai achieves FBBC through a collaborative workflow between the user and the application, as illustrated in the activity diagram (Fig. 1). The user makes key decisions at various stages, while the application performs computations, saves intermediate results, and presents relevant options based on those decisions. In this section, we present a concrete example of the FBBC process based on this workflow.

### Installing behavior Senpai

**Fig. 2:**
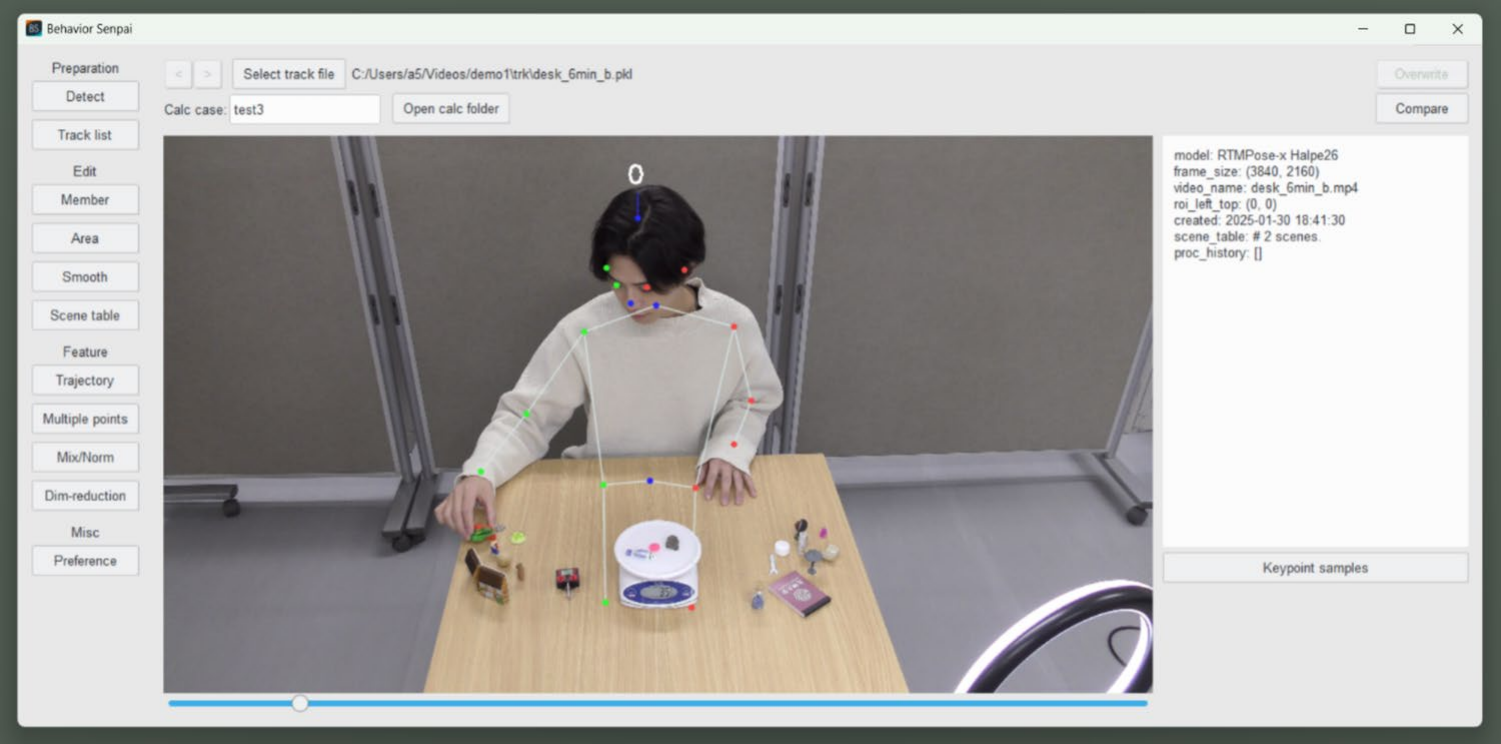
Main window of Behavior Senpai. *Note.* This screenshot shows the main window of Behavior Senpai. The buttons on the left give access to all the functions necessary for FBBC, including keypoint detection, while the center of the window shows the results of keypoint detection

FBBC utilizes dimensionality reduction in Behavior Senpai version 1.5 (Nishimura, 2025). As an application supporting FBBC, Behavior Senpai was developed in Python and is distributed under the GNU Affero General Public License version 3, which allows free use of the software. It is available at https://hdl.handle.net/2324/7160651. To install Behavior Senpai on Windows, users need to execute the BehaviorSenpai.exe file. Upon successful installation, a window similar to Fig. 2 appears, providing access to various FBBC functionalities.

### Folder structure

**Fig. 3.**
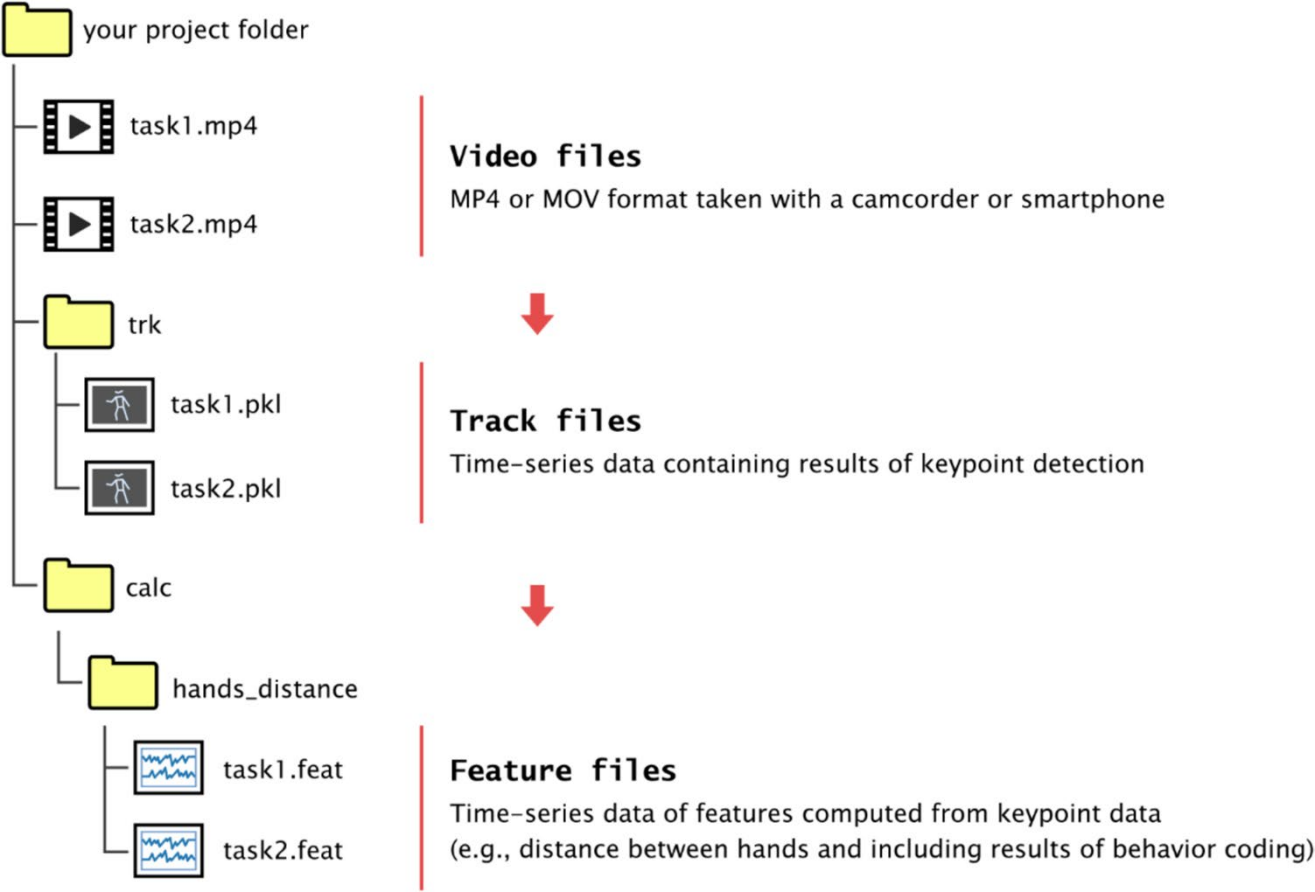
Folder structure and file types of Behavior Senpai

Behavior Senpai stores keypoint detection results as time-series data in binary files called “track files” that are saved in a “trk” folder created in the same location as the video file. Time-series data obtained from calculations, such as the distances between keypoints, are also saved in binary files similar to the track files. These files are referred to as “feature files” and are stored in a “calc” folder created in the same location as the video file. Because multiple feature files may be generated depending on the type of calculations performed, they are managed in subfolders within the “calc” folder. The names of these subfolders are defined by the user. Fig. 3 shows an example of the folder structure. For video files “task1.mp4” and “task2.mp4,” there are corresponding track files, and feature files are created in their respective subfolders.

## User interface

### Keypoint detection

**Fig. 4:**
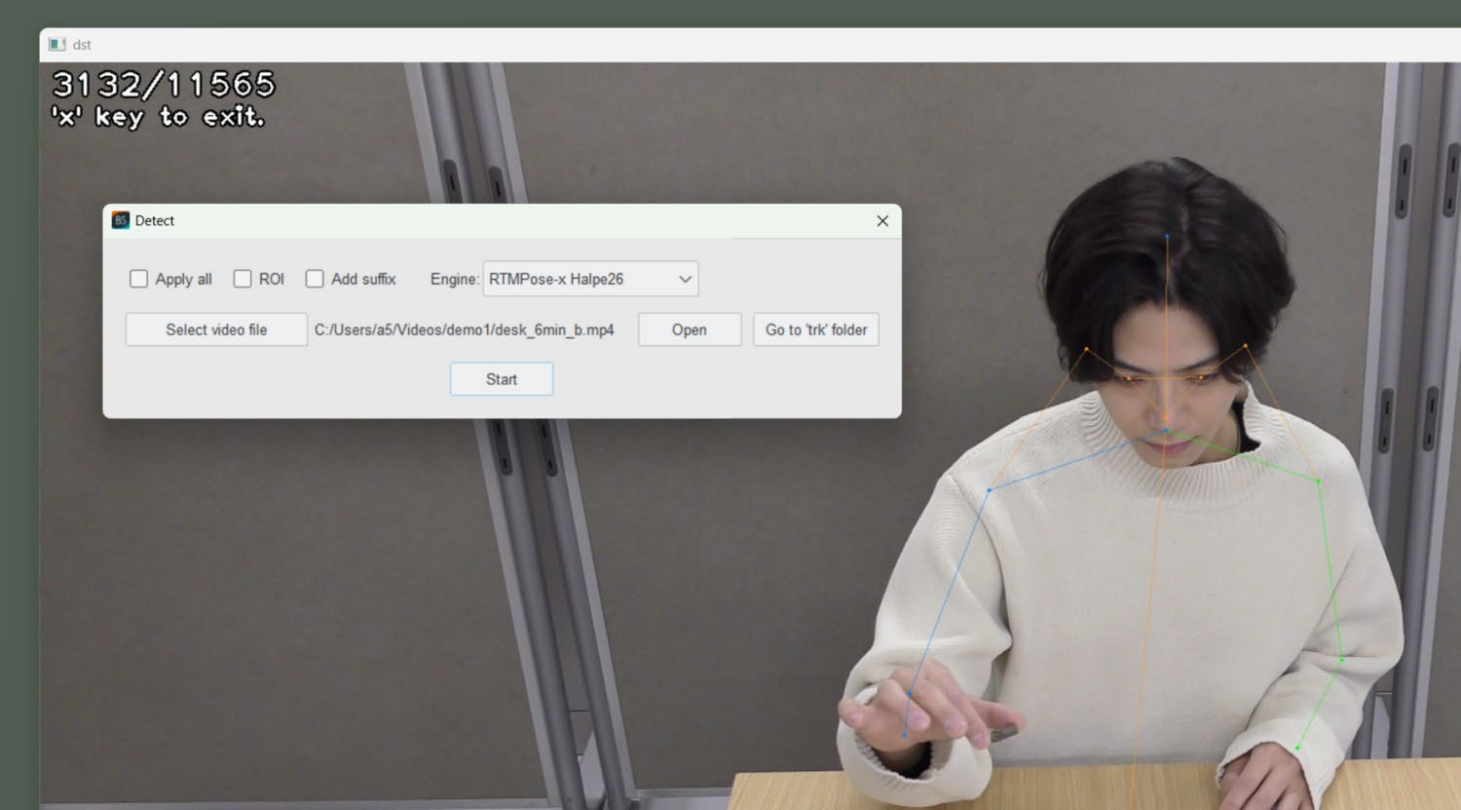
Keypoint detection in Behavior Senpai. *Note.* This screenshot is from the “Detect” window of Behavior Senpai. The user selects the video to be observed and the backend for keypoint detection, then they click “Start” to begin processing. Behavior Senpai has two optional functions for keypoint detection. One is the ability to target keypoint detection within a specified region (ROI: region of interest) of the video, and another is the option to apply keypoint detection to all files in a folder. All results are saved in the “trk” folder

**Fig. 5:**
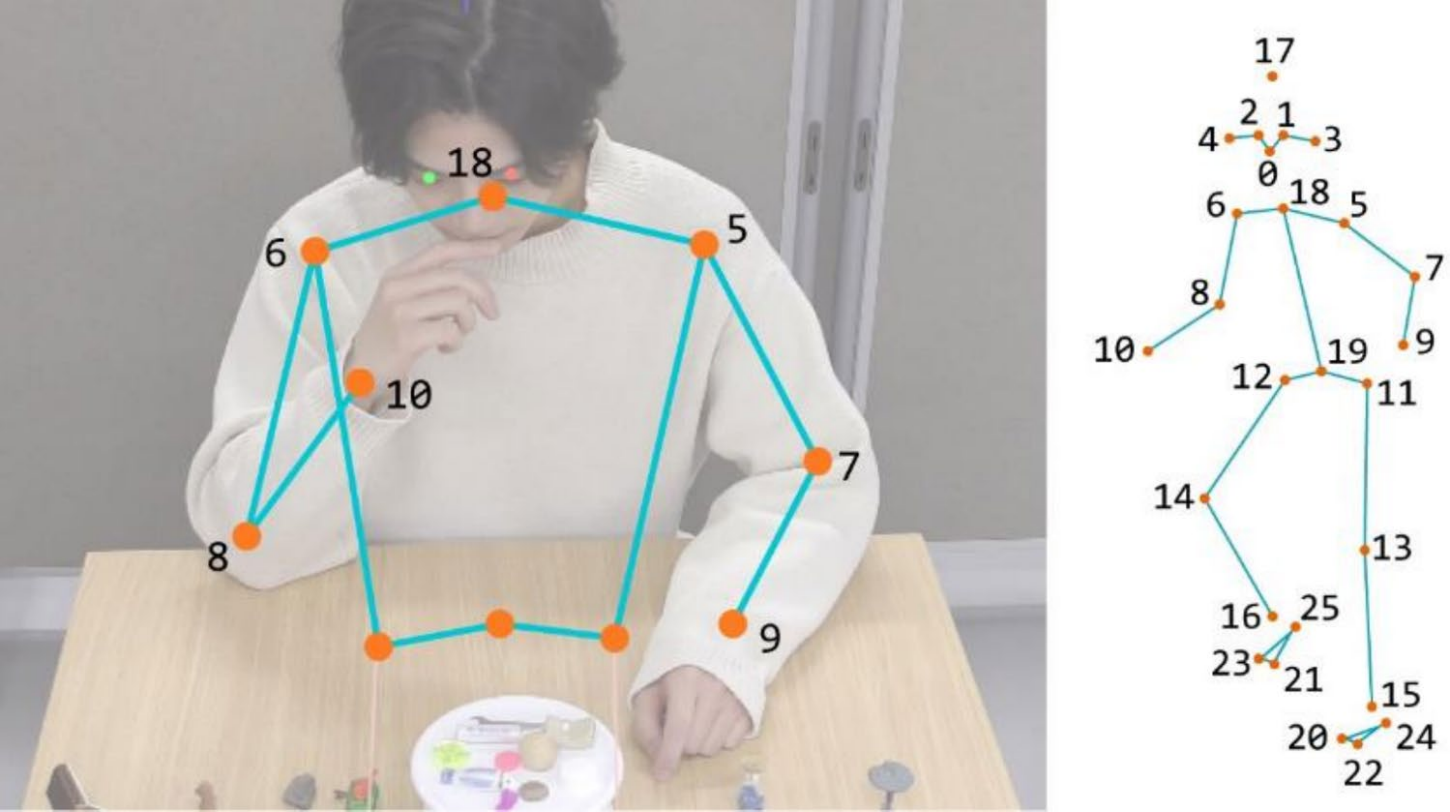
Keypoints of upper body detected by RTMPose-Halpe26 Model

Behavior Senpai enables researchers to perform FBBC interactively and visually. The first step is to execute keypoint detection and create the track file in the window shown in Fig. 4. Behavior Senpai supports MediaPipe Holistic by MediaPipe (Lugaresi et al., 2019), YOLO v8 (Jocher et al., 2024) and YOLO 11 (Jocher et al., 2025) by Ultralytics, and RTMPose by MMPose (Jiang et al., 2023) as backends for keypoint detection. YOLO and MMPose also support multi-person keypoint detection, allowing for the analysis of interactions between multiple individuals. For RTMPose, Halpe26 (Fang et al., 2023) is used as shown in Fig. 5.

### Support for DeepLabCut keypoint data

To facilitate the use of keypoint detection results from DeepLabCut, Behavior Senpai provides functionality to import and process its output files. DeepLabCut stores keypoint detection results in an HDF5 format, specifically as.h5 files. Behavior Senpai can parse these DeepLabCut keypoint data (.h5) files and convert them into a format compatible with its analysis framework.

With this functionality, feature-based behavior coding (FBBC), as proposed in this study, can be applied not only to keypoint detection results obtained through Behavior Senpai’s built-in methods but also to those generated by DeepLabCut. This extension allows researchers to conduct FBBC on a wider range of behavioral data, including both human and animal movements, thereby broadening its applicability in behavioral research.

### Scene definition

**Fig. 6:**
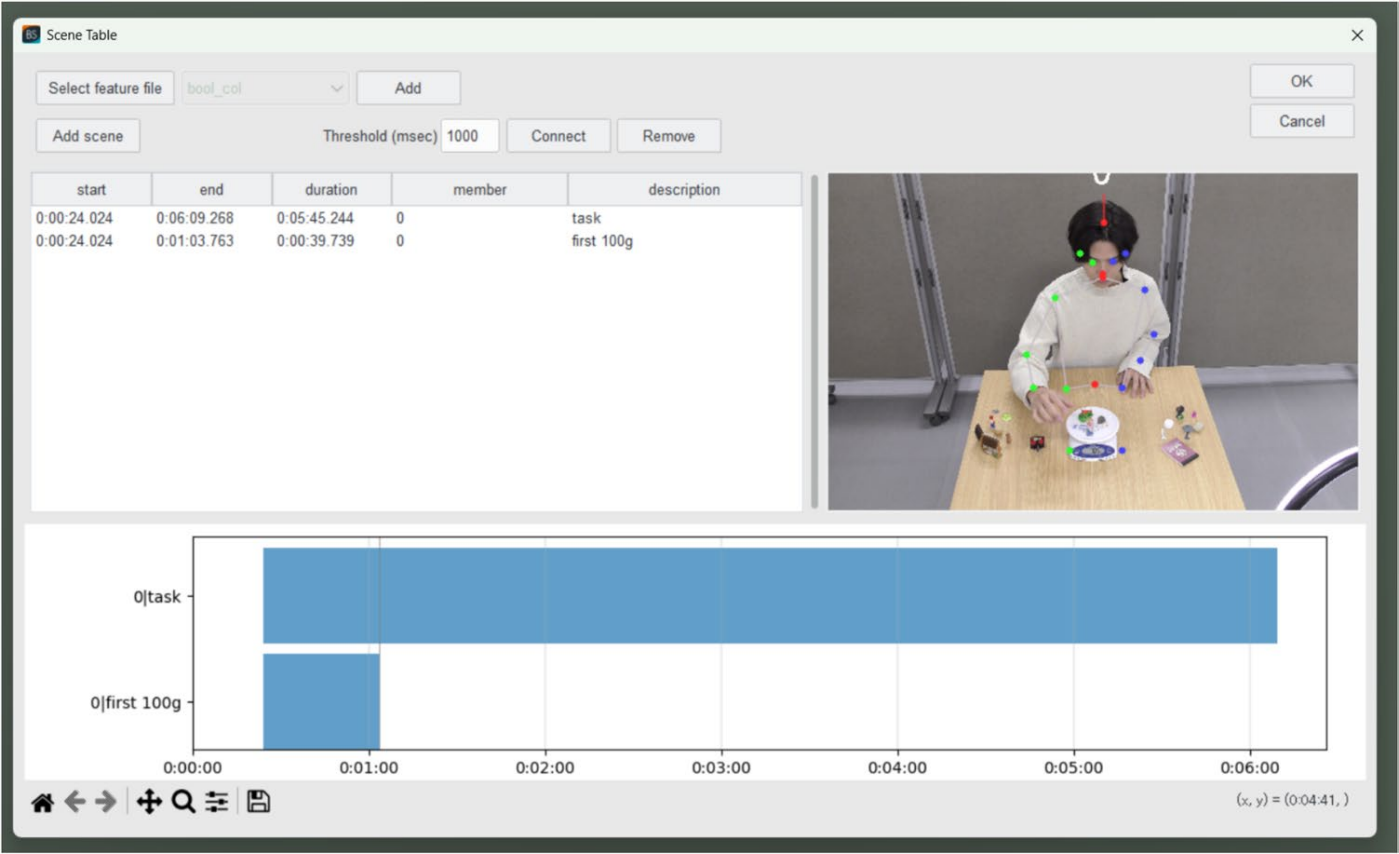
Scene definition in Behavior Senpai. *Note.* This screenshot shows the “Scene Table” window of Behavior Senpai. To perform FBBC, the user inputs the desired time range and description and then clicks the “Add scene” button to generate a graph. Interaction with the graph is enabled; clicking on it displays the corresponding video frame and copies the timestamp to the clipboard. These actions are finalized by clicking the “OK” button

In many cases, video files include scenes before and after the subject performs the task, and because these do not require coding, it is desirable to exclude them beforehand. Behavior Senpai provides a function for defining the scenes that are necessary for FBBC. This function is also useful when the user wishes to perform more detailed coding for specific sets of behaviors during the task. As shown in Fig. 6, users can define scenes in which the subject is performing tasks, or they can specify particular time periods.

### Featurization with multiple keypoints

Via its “Trajectory” window, Behavior Senpai offers functionality for calculating and outputting features such as the (*x*, *y*) positions of keypoints and their displacements per unit time. Also, from the “Multiple points” window, users can compute the relative positions of arbitrary keypoints. The options for calculating positional relationships include not only the distance between two points but also the dot product and cross product of vectors formed by three points. Postprocessing the feature files obtained from the “Trajectory” and “Multiple points” windows, such as normalization, can improve the results of dimensionality reduction. Behavior Senpai provides functions for adding and dividing multiple features, as well as normalization and binarization, via the “Mix/Norm” window, which also allows for the concatenation of multiple feature files.

### Dimensionality reduction

Having established the methods for feature extraction and processing in Behavior Senpai, we turn to a crucial step in behavioral coding: dimensionality reduction. This is particularly valuable when dealing with the complex multidimensional data generated by feature extraction. Scatter-plotting multiple features in a plane after dimensionality reduction is a powerful tool for data analysis. High-dimensional data can be challenging to interpret, but dimensionality reduction transforms them into two dimensions, making the data visually comprehensible. This allows for the easy identification of patterns, clusters, and outliers that may be hidden in higher dimensions. Also, dimensionality reduction helps to remove irrelevant noise, thereby preserving essential information and highlighting the data’s intrinsic structure. This results in improved clarity and accuracy for subsequent analyses.

In Behavior Senpai, dimensionality reduction is performed using uniform manifold approximation and projection (UMAP) (McInnes et al., 2018). Compared to traditional methods such as principal component analysis (PCA), UMAP is better suited for preserving local structures within high-dimensional feature spaces, making it advantageous for visualizing behavior-related data. This is particularly beneficial for behavioral data, especially posture data, which contains complex spatial relationships between numerous keypoints with often nonlinear similarity characteristics. In the exploratory phase of FBBC, it is crucial that similar posture patterns are visually distinguishable in low-dimensional space. UMAP excels at this by maintaining local distance relationships while also considering global structure, resulting in greater capacity to spatially position similar postures close together, which facilitates pattern discovery. Additionally, UMAP offers superior computational efficiency compared to other nonlinear dimensionality reduction methods like t-distributed stochastic neighbor embedding (t-SNE) (McInnes et al., 2018), providing practical advantages when processing large volumes of behavioral data. By specifying which features and parameter values to use for UMAP, users can easily create scatter plots. Furthermore, dimensionality is reduced to two rather than three dimensions to prioritize usability in the user interface, ensuring intuitive data exploration and visualization. The UMAP parameters that impact the results the most are “N_neighbors” and “Min_dist,” and it is practical to try multiple values of these parameters in order to search for well-separated results. Empirically, it has been found that in most cases, good results are obtained with N_neighbors = 20 or 50 and Min_dist = 0.1. To obtain well-separated scatter plots, it is also important to thin out the UMAP features. Empirically, it has been observed that such thinning tends to facilitate clustering by removing transitional frames, that is, intermediate postures between one posture and another. Since the appropriate amount of thinning depends on factors such as how much rapid movement is involved in the task, it is practical to determine this amount via exploration. To that end, Behavior Senpai allows the interactive adjustment of parameters.

**Fig. 7:**
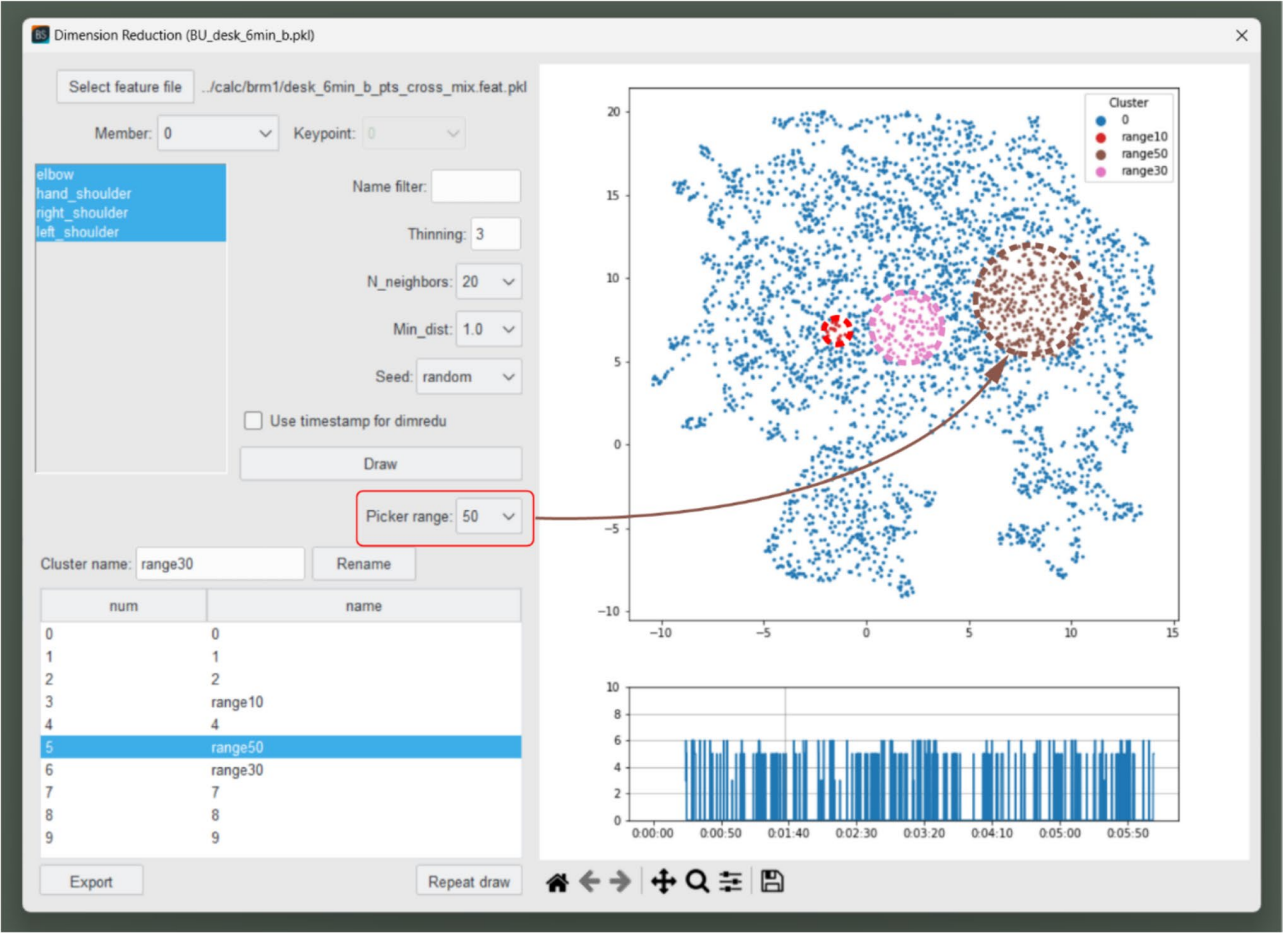
Manual clustering on scatter plot via mouse clicks with a specified picker range (10, 30, or 50). *Note.* This screenshot shows the “Dim-reduction” window of Behavior Senpai. The user can set parameters that affect the UMAP results, select features, and thin out the time-series data. Left-clicking on a point in the scatter plot displays the video frame corresponding to that point. Clustering is performed manually by the user. By selecting a cluster from the table in the bottom left and then right-clicking on the scatter plot, the selected points are assigned to that cluster, with the right-click range specified via “Picker range.”

Users can then perform clustering while confirming visually with mouse clicks which video frames correspond to which parts of the scatter plot, as shown in Fig. 7. These steps achieve FBBC with dimensionality reduction.

## A workflow example

### Target video and its overview

**Fig. 8:**
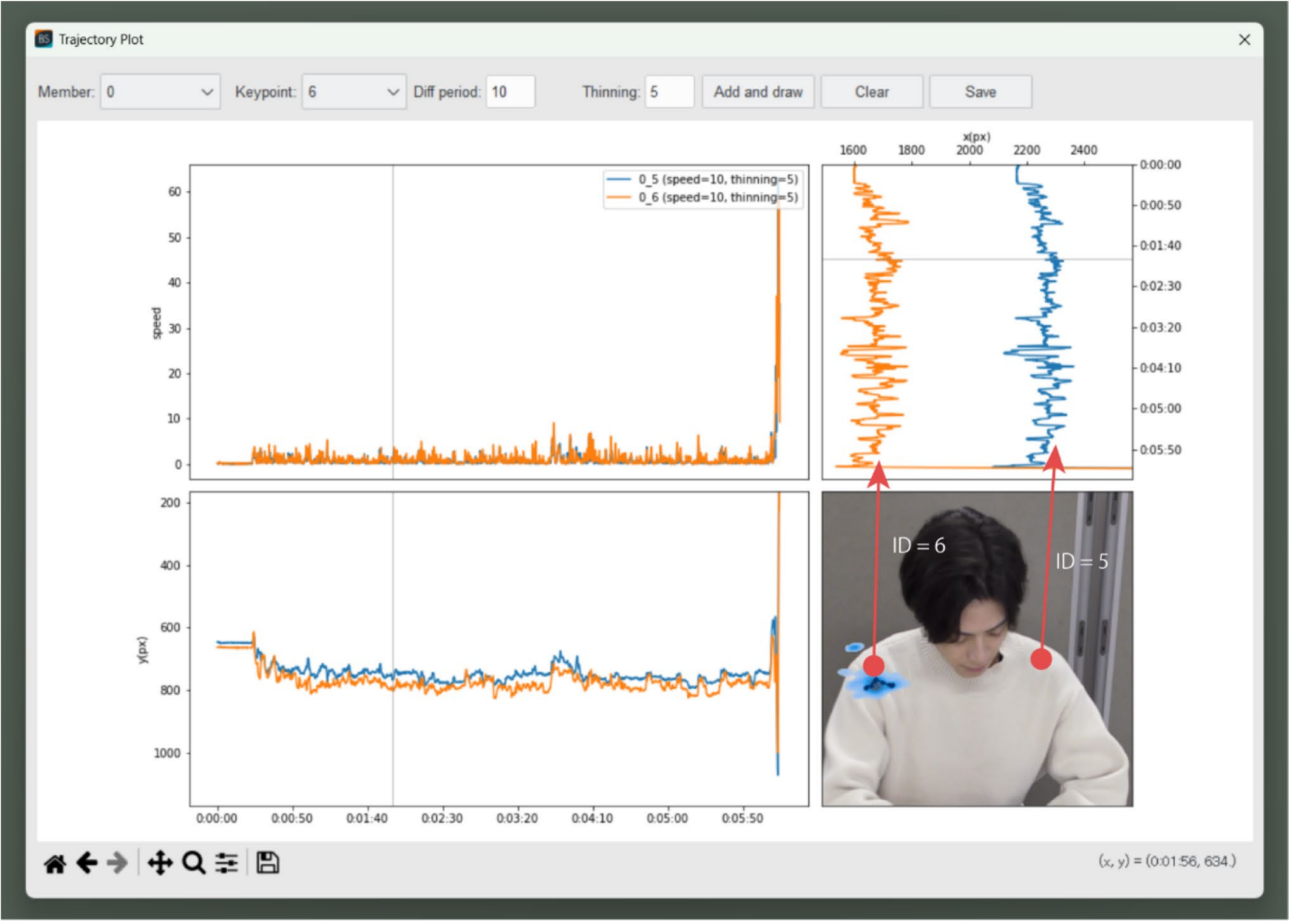
Time-series data for (x, y) coordinates of shoulders (ID = 5, 6) from Behavior Senpai. *Note.* This screenshot is of the “Trajectory” window of Behavior Senpai, in which the time-series data of the (*x*, *y*) coordinates of the shoulder keypoints (ID = 5, 6) are plotted as line graphs. The instantaneous speeds of the keypoints are plotted in the upper left. In the lower right, the degree to which the right-shoulder keypoint stayed still in the video is plotted on the video frame using the kernel density estimate (KDE). Darker blue indicates that the keypoint stayed at the location for longer. This visualization shows that the upper body moved from side to side during the task

**Fig. 9:**
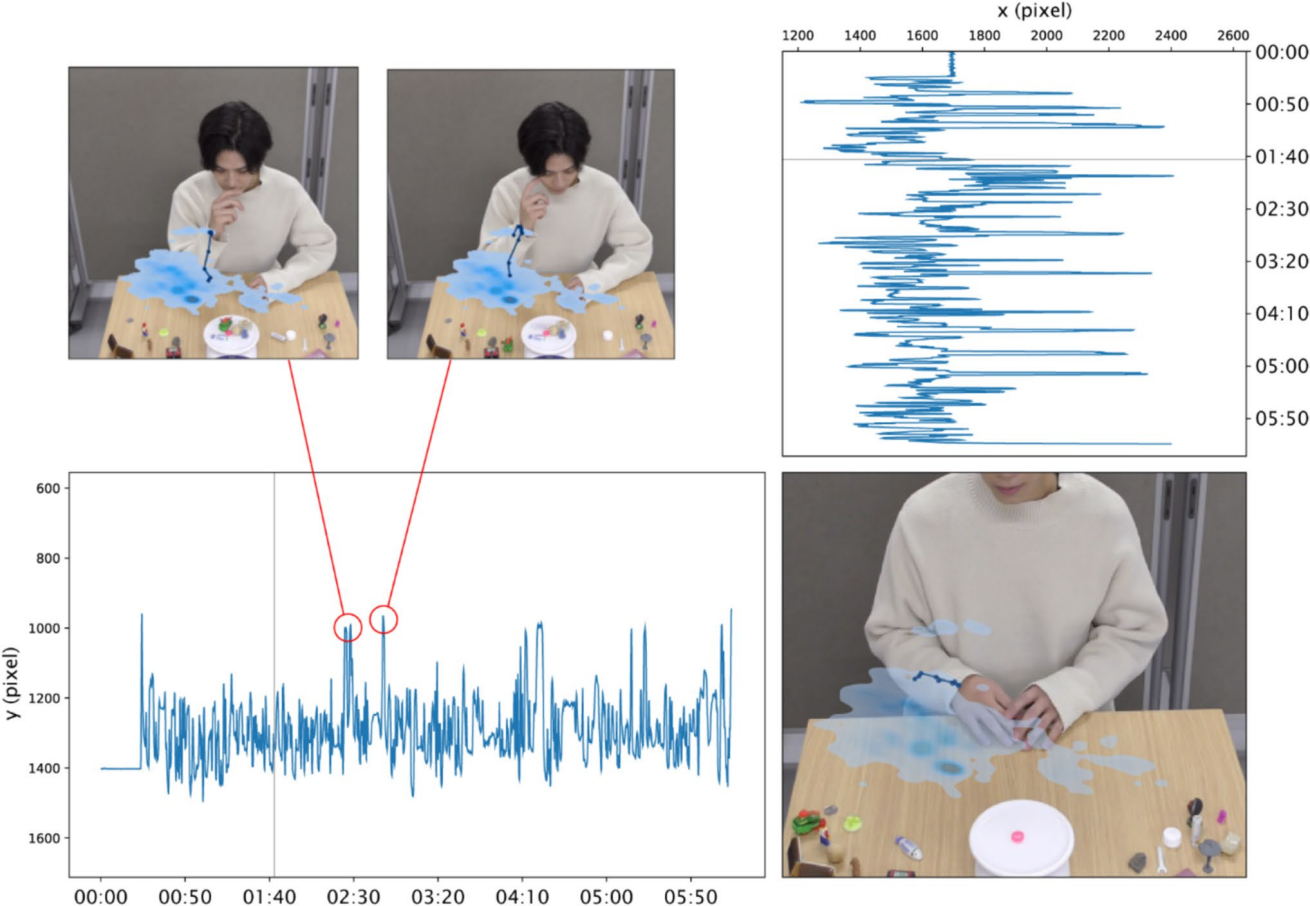
Time-series data for (x, y) coordinates of right hand (ID = 10)

In this paper, we demonstrate the operation of FBBC with a video entitled “desk_6 min_b.mp4” (Nishimura, 2024). The video shows a subject placing various objects on and off a scale so that the latter reads exactly 100 g. The video duration is 6 min 25 s at a frame rate of 29.97 fps. After keypoint detection,

Figure 8 shows the trajectories of both shoulders (ID = 5, 6), and Fig. 9 shows the trajectory of the right hand (ID = 10). The shoulder trajectories clearly show that the whole upper body is moving left and right. Also, the subject is mainly using his right hand and is reaching for an object that is on the left side of his body. Furthermore, in some scenes, the subject lifts his right hand close to his face, possibly as an unconscious or habitual movement, such as briefly touching his chin or scratching his cheek. Since this gesture occurs for only a very short duration in the video, it is easily missed. The red circles in Fig. 9 highlight the corresponding points in the trajectory where these movements are observed.

### Featurization

**Fig. 10:**
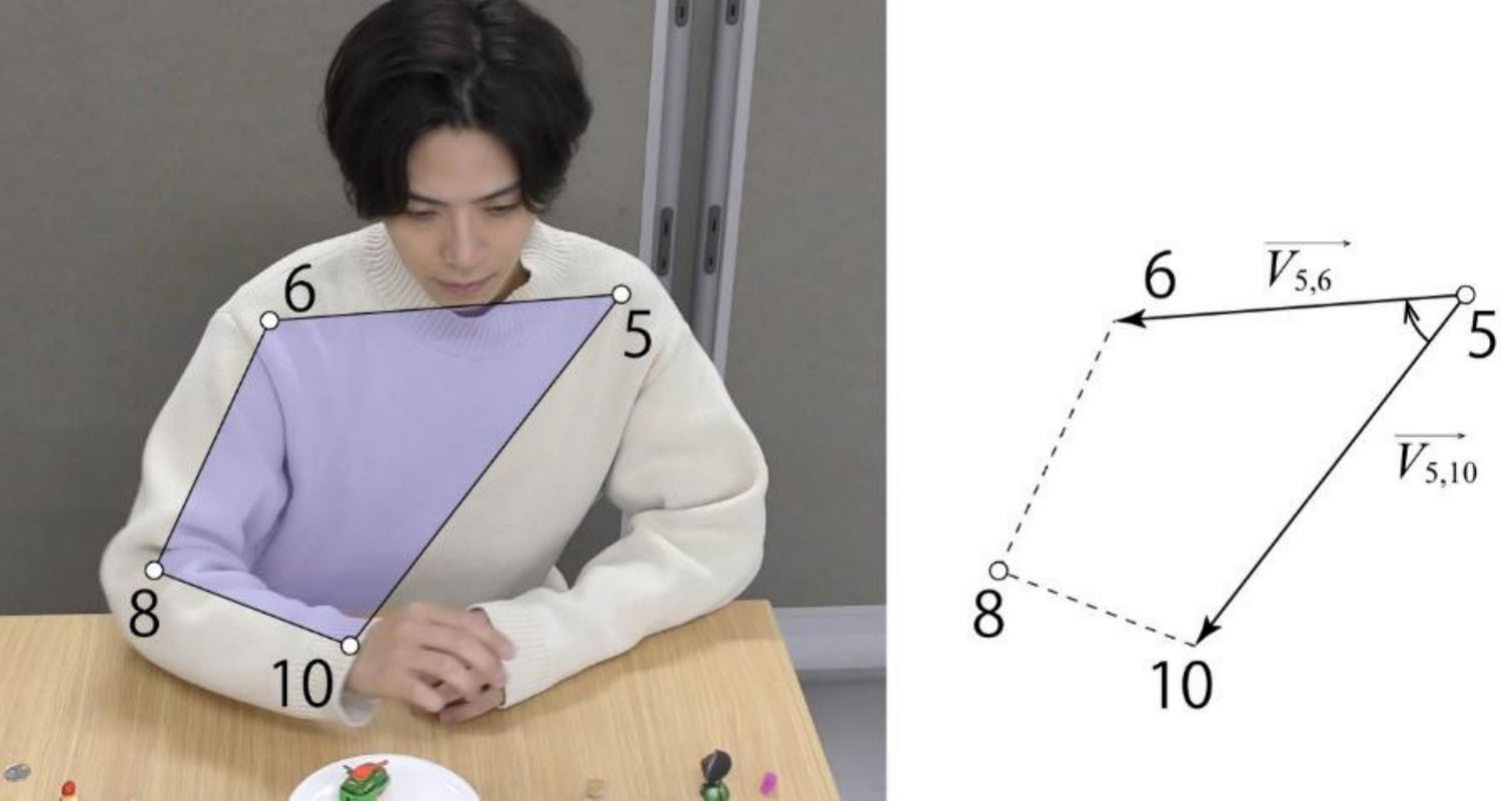
Featurization of posture of right arm by cross products. *Note.* Since the origin of the image is at the top left, the cross product is handled using a left-handed coordinate system in Behavior Senpai

**Fig. 11:**
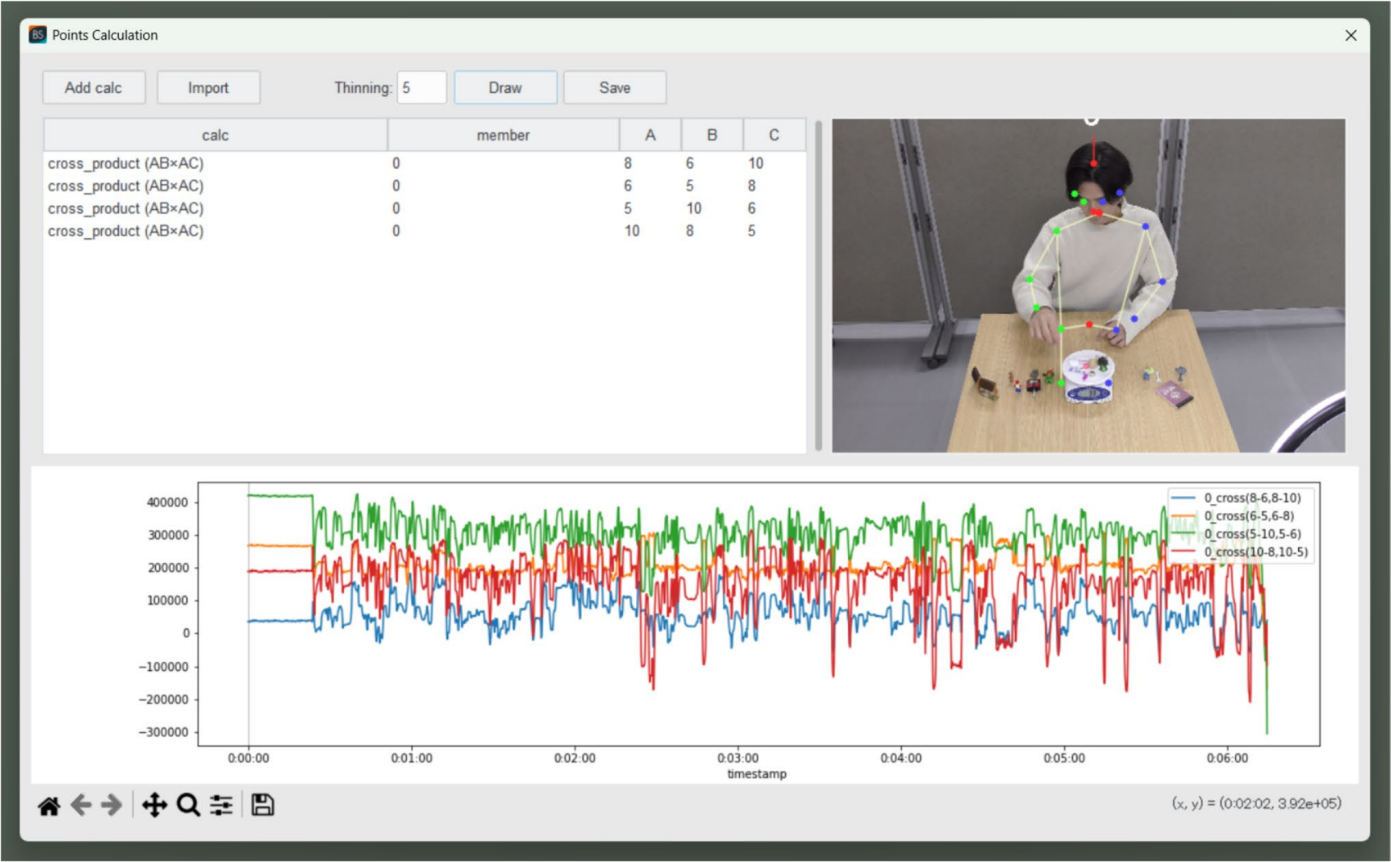
Calculation and results of four cross products. *Note.* This screenshot is of the “Multiple points” window of Behavior Senpai. The calculation results are obtained as time-series data by specifying the IDs of the keypoints to be calculated and the calculation method, such as “cross_product.”

In this example, for feature extraction, we focus on changes in the posture of the right arm and the left–right movement of the upper body. Specifically, we use the *x*-coordinate of the neck (ID = 18) as a feature for the upper-body movement, and we use the relative positions of multiple keypoints related to the right arm as features for the right-arm posture. The cross product of vectors formed by three keypoints can be interpreted as a feature representing the relative positions of those keypoints, and as features, we used four cross products calculated from four keypoints (ID = 5, 6, 8, 10) as shown in Fig. 10. The results displayed by Behavior Senpai are shown in Fig. 11.

$$\begin{array}{l}{F}_{shoulde{r}_{left}}=\overrightarrow{{V}_{\text{5,10}}}\times \overrightarrow{{V}_{\text{5,6}}}\\ {F}_{hand}=\overrightarrow{{V}_{\text{10,8}}}\times \overrightarrow{{V}_{\text{10,5}}}\\ {F}_{shoulde{r}_{right}}=\overrightarrow{{V}_{\text{6,5}}}\times \overrightarrow{{V}_{\text{6,8}}}\\ {F}_{elbow}=\overrightarrow{{V}_{\text{8,6}}}\times \overrightarrow{{V}_{\text{8,10}}}\end{array}$$

**Fig. 12:**
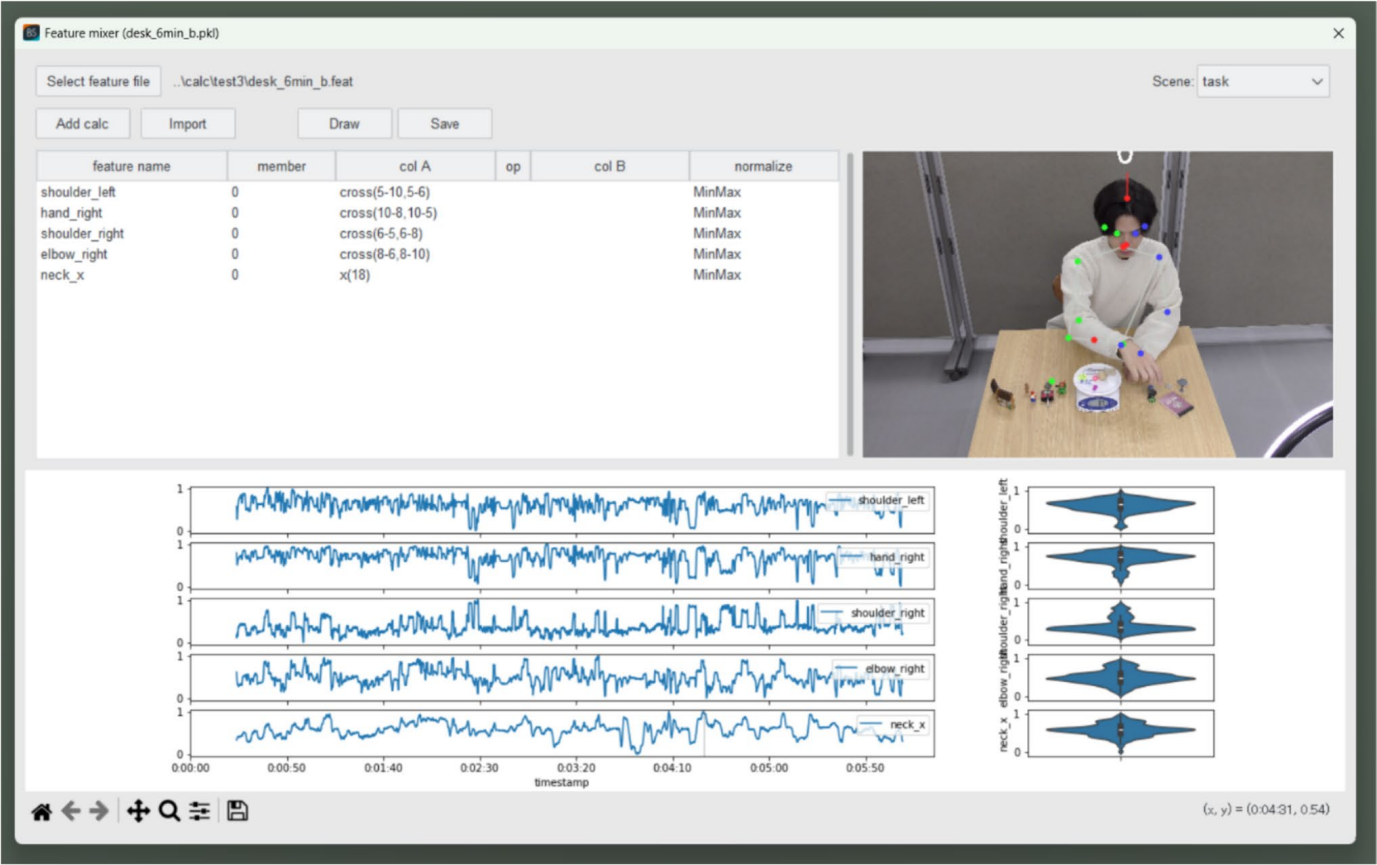
Min–max normalization of features. *Note.* This screenshot is of the “Mix/Norm” window of Behavior Senpai. Users can concatenate multiple feature files and perform normalization and basic arithmetic operations (addition, subtraction, multiplication, and division) on each feature

To create the final feature set, we concatenate the *x*-coordinate data for the neck (ID = 18) with the four cross products obtained earlier, and we use these data after individual min–max normalization as features of the behavior during the task. Users can perform these operations in the “Mix/Norm” window as shown in Fig. 12. We normalize the features because if the range of values for each feature varies greatly, then the UMAP results become more susceptible to the influence of specific features.

### Behavior coding

**Fig. 13:**
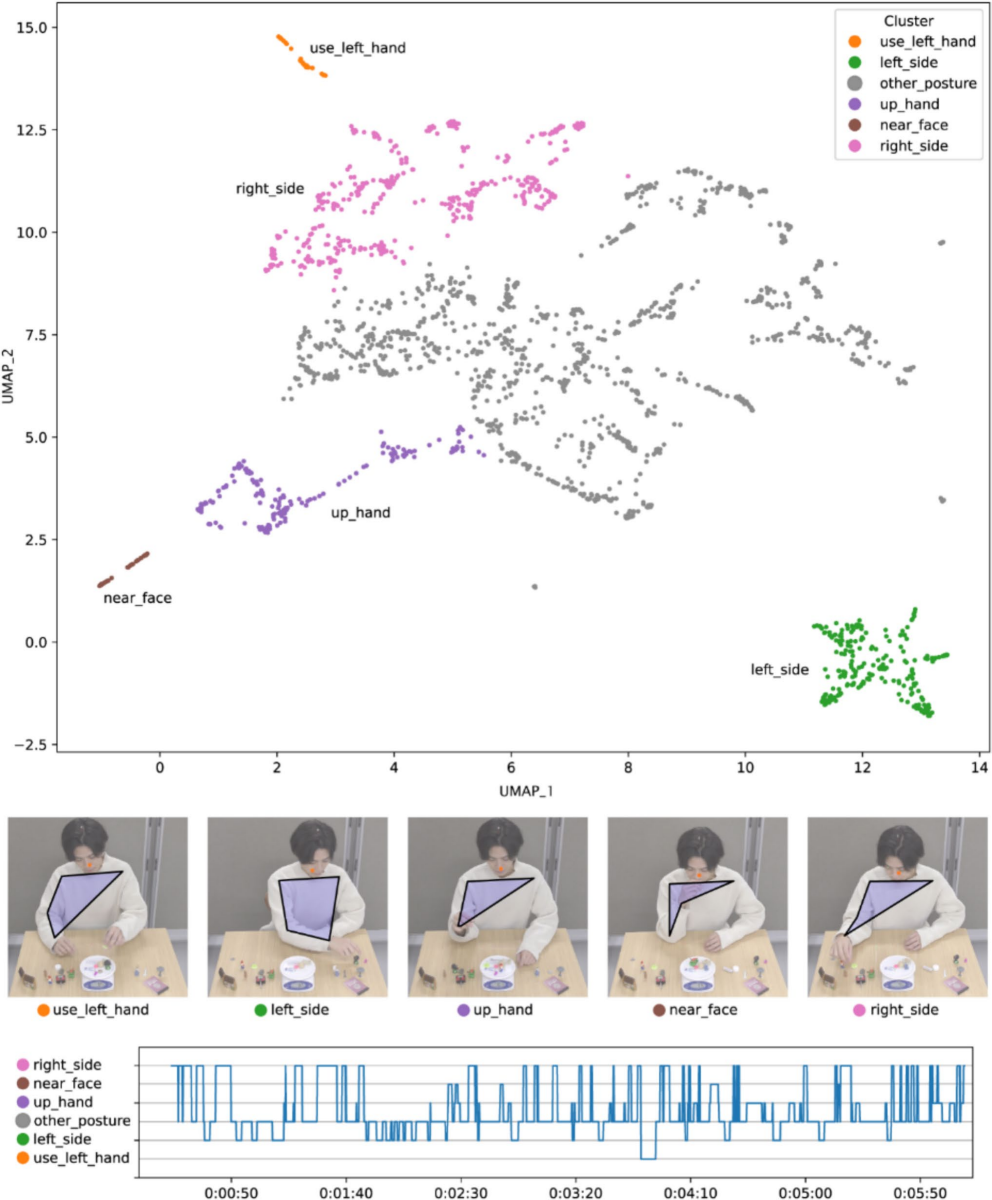
Results of clustering and behavior coding

Figure 13 shows the results of thinning the created features by five frames (equivalent to about 0.17 s), performing dimensionality reduction using UMAP, and clustering. The UMAP parameters were set after exploration to achieve well-separated scatter plots, with N_neighbors = 30 and Min_dist = 0.1.

In this example, five postures were clearly classifiable: “reaching to the left side” (left_side), “raising the hand while resting on the elbow” (up_hand), “putting the hand near the face” (near_face), “reaching to the right side” (right_side), and “using left hand” (use_left_hand). Although the left hand (ID = 9) was not included in the features, clustering was possible because of the characteristic posture of the right arm when using the left hand. The lower part of Fig. 13 shows a graph of the clustering results as time-series data. This is the result of behavior coding in FBBC.

### Observation

Of the five obtained behavioral states, “up_hand,” “near_face,” and the other states in which the hand is not reaching for the object or the scale can be interpreted as codes for thinking or hesitating. Also, these states occur more frequently during the time interval of 2:30–5:00. In this way, the FBBC results offer a foundation for developing coding schemes.

## Conclusions

This paper has demonstrated the feasibility of complex posture classification by combining multiple features, dimensionality reduction, and manual clustering. However, while highlighting the potential of this approach, our study has also identified several limitations and future challenges that warrant further investigation.

A primary challenge is feature design. The absence of a universal method for expressing desired postures or behaviors necessitates experimentation with various combinations of keypoints and computational methods. To address this challenge, we propose developing feature presets suitable for diverse observational contexts, based on accumulated case studies.

The current iteration of our proposed method is limited to classifying instantaneous “postures” and does not extend to actions involving keypoint movements over time. Despite this limitation, we believe that the posture classification method presented in this study contributes considerably to the expansion of behavior coding capabilities. Behavior Senpai includes functionality for calculating keypoint movement over time, which could serve as a foundation for future action classification efforts.

Another limitation lies in the dimensionality reduction process. In its current implementation, the reduction is fixed to two dimensions, which may constrain the classification of more complex feature sets. Allowing three-dimensional embeddings could potentially improve the differentiation of intricate behavioral features. Additionally, UMAP is the only dimensionality reduction method currently supported. Exploring alternative techniques, such as t-SNE (Maaten & Hinton, 2008), may further enhance the flexibility and effectiveness of FBBC in capturing behavioral nuances.

A further limitation relates to multi-person tracking reliability. While both YOLO and MMPose support multi-person keypoint detection, they have tracking constraints that impact interaction analysis. MMPose primarily identifies individuals based on spatial position, which means that person IDs may change when individuals overlap or exchange positions. YOLO maintains ID consistency within frames but may assign new IDs when a person exits and re-enters the frame. These limitations are particularly important to consider when analyzing long-term interactions between multiple individuals.

FBBC offers the flexibility to focus on specific body parts, making it particularly valuable in reducing time-consuming tasks during the exploratory phase of behavior definition. FBBC and its implementation in Behavior Senpai represent an advancement in behavioral research, especially for exploratory studies. By bridging traditional behavioral coding methods with modern machine-learning techniques, FBBC provides researchers with a powerful tool for analyzing diverse postural patterns. The strengths of FBBC, as demonstrated through Behavior Senpai, include improved efficiency via feature time-series conversion and enhanced flexibility in coding scheme development. Crucially, FBBC addresses the long-standing challenge of repetitive observations in traditional behavioral coding methods.

The FBBC approach synergizes automated feature extraction with human insight, leveraging computational efficiency and nuanced human judgment. While the current focus is on keypoint detection and clustering, the FBBC framework holds potential for further expansion, possibly incorporating more advanced machine-learning techniques in future iterations. It has been reported that for relatively simple behaviors in animals such as mice, automated classification of behaviors using keypoint-MoSeq (Weinreb et al., 2024) is possible. While it is currently unclear whether human behaviors can be automated using similar methods in the future, we believe that the insights gained through FBBC have a high potential to contribute to the automation of coding. This potential for future automation underscores the significance of FBBC in advancing behavioral research methodologies, bridging the gap between traditional observational techniques and emerging computational approaches.

In conclusion, FBBC as implemented in Behavior Senpai marks a notable advancement in behavioral research. By addressing key limitations of traditional coding methods while embracing modern technology, FBBC has the potential to transform researchers’ approaches to studying complex human behaviors. As this method is adopted and refined, we anticipate that it will contribute to more efficient, comprehensive, and insightful behavioral analyses, ultimately advancing our understanding of human behavior across the psychological and behavioral sciences.

## Data Availability

The sample video file used in this article can be accessed at 10.48708/7172619.

## Code availability

Behavior Senpai version 1.5, the software used in this study, can be freely downloaded from https://hdl.handle.net/2324/7160651. The DOI reference for this software is 10.48708/7160651.
